# UTROSCT: a case report and scoping review of ultrasound features, with exploratory serum proteomic findings

**DOI:** 10.3389/fonc.2026.1892074

**Published:** 2026-08-24

**Authors:** Davide Castelli, Ghergana Alexandrova Topouzova, Blendi Ura, Francesca Buonomo, Eduardo Maria Sommella, Alessandro Mangogna, Federico Romano, Giovanni Di Lorenzo, Stefania Cicogna, Andrea Romano, Giuseppe Ricci

**Affiliations:** 1 Department of Medicine, Surgery and Health Sciences, University of Trieste, Trieste, Italy; 2 Institute for Maternal and Child Health, IRCCS Burlo Garofolo, Trieste, Italy; 3 DIFARMA Department, University of Salerno, Salerno, Italy; 4 Pathology (UCO Anatomia Patologica), Cattinara Teaching Hospital, Trieste, Italy

**Keywords:** case reports, proteomics, scoping reviews, sex cord-gonadal stromal tumors, ultrasonography, uterine neoplasms

## Abstract

**Background and objectives:**

UTROSCT is a rare uterine neoplasm with nonspecific imaging features. The aim of this study was to evaluate how UTROSCT is described on pelvic and transvaginal ultrasound in the published case-report literature and to report the clinicopathologic, immunophenotypic, and exploratory serum proteomic features of a new UTROSCT case.

**Case presentation:**

Transvaginal ultrasound revealed an intracavitary uterine lesion in a 72-year-old asymptomatic postmenopausal woman. Histopathology and immunohistochemistry on biopsy and hysterectomy specimens supported the diagnosis of group II UTROSCT with no myometrial invasion and no lymph node involvement. Exploratory serum proteomic analysis, performed as a hypothesis-generating approach, suggested enrichment of extracellular matrix–related proteins compared with a healthy control and an endometrial stromal sarcoma control.

**Materials and methods (scoping review):**

We searched MEDLINE (PubMed), Scopus, and Google Scholar from January 2004 to December 2025 to identify English-language case reports of histologically confirmed UTROSCT that included at least one pelvic/transvaginal ultrasonographic assessment. Records were screened independently by two reviewers. The review was conducted and reported according to PRISMA ScR. Data were charted using a predefined form capturing age and ultrasound presentation categories (e.g., fibroid-like, polyp-like, pelvic mass/unspecified). Reporting quality was appraised using the JBI Critical Appraisal Checklist for Case Reports; appraisal results were not used for exclusion.

**Results (scoping review):**

Out of 359 case reports identified, only 99 were included in the evidence mapping. The mean age of the examined population is 49.2 years. The results of our review showed that UTROSCT presents as a fibroid in the majority of cases (46.5%).

**Discussion and conclusions:**

Diagnosis of UTROSCTs could be challenging because it is a rare condition. The ultrasound appearance is heterogeneous and often not accurately reported in literature. Our review shows that UTROSCT most frequently mimics leiomyoma at ultrasound. In our case report, exploratory serum proteomics may generate hypotheses for future translational research but requires tissue-level validation.

## 1 Introduction

Uterine Tumors resembling Ovarian Sex Cord Tumors (UTROSCT) are rare tumors (<1% of uterine mesenchymal tumors). According to the World Health Organization (WHO), UTROSCTs are tumors of uncertain behavior with low malignant potential, originating from pluripotent mesenchymal, endometrial stromal, or displaced ovarian sex cord cells (1).

Currently, due to the rarity of UTROSCTs, there are limited data available regarding its epidemiology. According to the European Cancer Information System, the incidence of uterine corpus cancers in Europe in 2022 was 5.68% (92,692 women per year) (2). According to the 2022 WHO data, mesenchymal tumors account for 8% of uterine corpus cancers (7,415 women per year), while UTROSCT accounts for <1% of uterine mesenchymal tumors (<74 women per year) (1). Based on Eurostat data published in 2024, the female population in Europe was 229 million as of January 2023, corresponding to an estimated annual incidence of UTROSCT of 0.00003% (approximately 1 case per 3 million women per year in Europe). These data highlight the rarity of the condition (3).

To date, there is no official classification of UTROSCT, and it is based on the one proposed in 1976 by Clement and Scully, who arranged it into 2 groups based on the percentage of sex-cord-like elements. Specifically Group I tumors composed partly (<50%) of sex-cord-like elements (endometrial stromal tumors with sex-cord-like elements; ESTSCLEs), and Group II tumors composed mainly (50–100%) of sex-cord-like elements (classic UTROSCTs). It is important to underline that Group I tumors are associated with recurrence and metastasis while Group II tumors appear to have a benign behavior (4).

UTROSCTs have a polyphenotypic immunohistochemical profile, with variable positivity for sex cordon markers (inhibin, calretinin, WT1, CD56, CD99, SF1, FOXL2 and melan-A), epithelial markers, estrogen receptor (ER), progesterone receptor (PR), smooth muscle markers (actin, desmin and h-caldesmon), and CD10. UTROSCTs are staged as uterine sarcoma using the FIGO, UICC and AJCC classifications (1).

Preoperative diagnosis is difficult because UTROSCT lacks pathognomonic imaging features. The most common treatment is total laparoscopic hysterectomy with or without bilateral salpingo oophorectomy. A conservative surgical approach may be considered in carefully selected patients, based on specific clinical criteria and individualized assessment. The recurrence rate is estimated to be around 5% (5, 6).

In this article, we conducted a literature review to analyze the main ultrasonographic features of UTROSCTs. To date no studies have specifically focused exclusively on the ultrasound features of UTROSCT and the available evidence is limited to individual case reports. Additionally, we present a case report of a 72-year-old patient with group II UTROSCT and an exploratory serum proteomic profile as a hypothesis-generating approach to explore a potential systemic phenotype related to extracellular matrix (ECM) remodeling associated with the tumor. At present, there is no robust evidence linking specific ultrasonographic patterns to serum proteomic signatures in UTROSCT; therefore, the proteomic findings are presented separately and interpreted cautiously.

## 2 Case report

The case report followed the CARE criteria for clinical case reporting.

**Figure 1:**
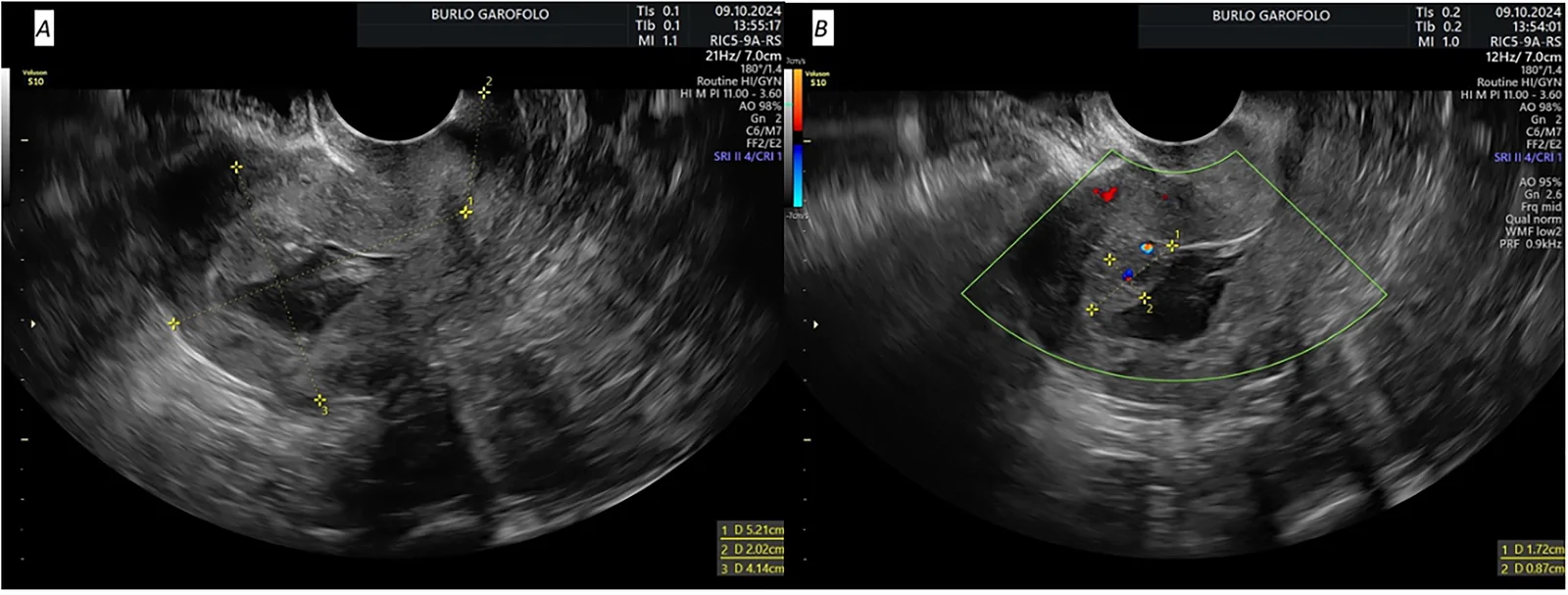
**(A)** Sonographic appearance of UTROSCT. **(B)** Measurements of the intracavitary lesion applying Power Doppler.

A 72-year-old healthy postmenopausal woman with a history of two pregnancies (two full-term live births) went to the gynecologist for a checkup. She was asymptomatic. There was no family history of gynecological cancer. Gynecological examination was normal. An office transvaginal ultrasound scan revealed an enlarged uterus with a thin endometrium distended by a modest amount of echogenic fluid and an intracavitary focal lesion located in the antero-fundal uterine wall. According to the IETA Consensus the lesion appeared heterogeneously hyperechoic, with regular margins, some hyperechoic foci and diffuse intralesional vascularization CS2 without an identifiable feeding vessel, measuring 17 × 8 × 25 mm (**Figures 1A, B**) (7).

**Figure 2:**
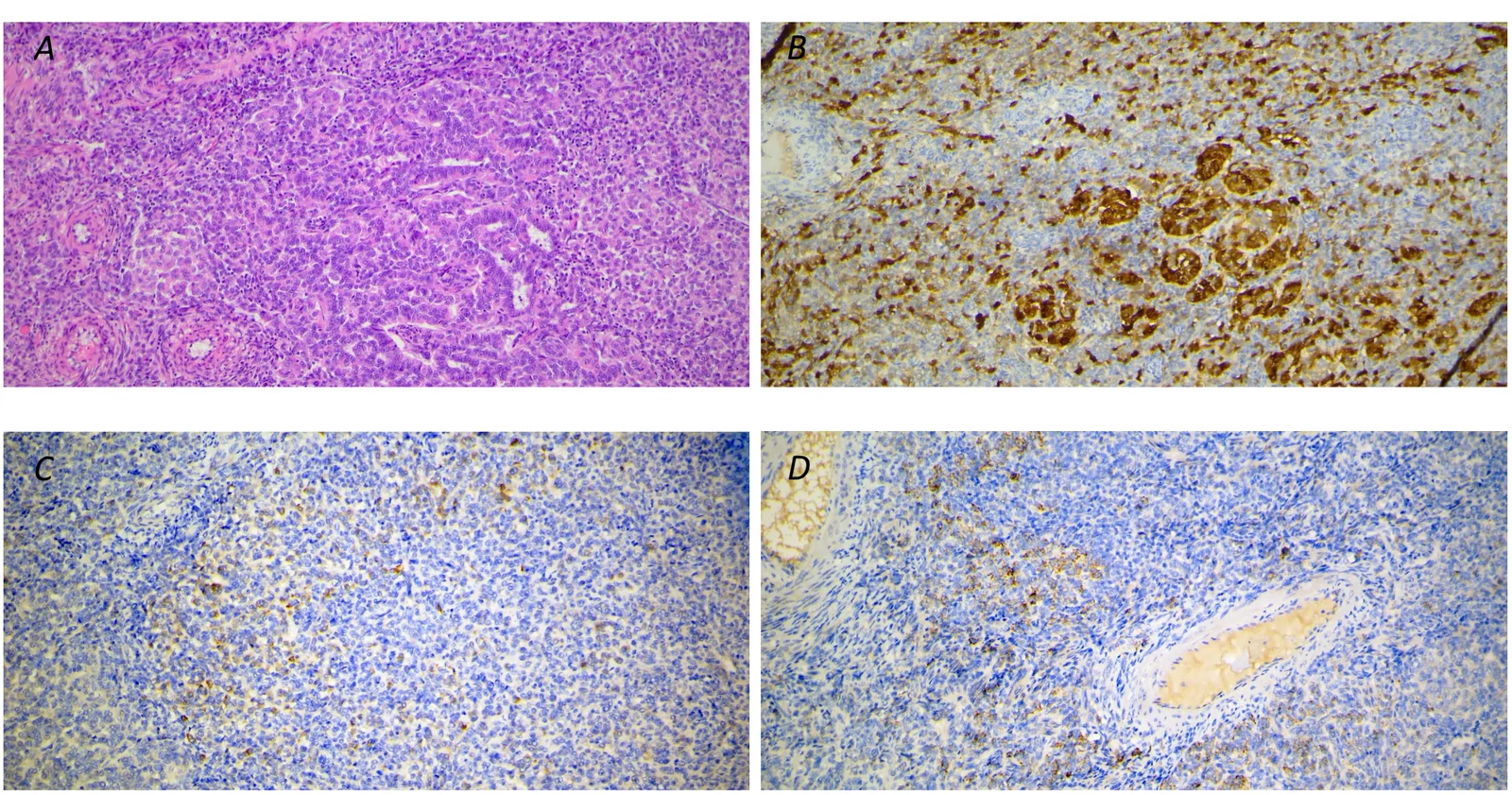
Histopathological and immunohistochemical features of uterine tumor resembling ovarian sex cord tumor (UTROSCT), group II. **(A)** Hematoxylin and eosin staining at 100× magnification highlights a small, well-demarcated tumor area composed of medium- to large-sized epithelioid cells arranged in densely packed nests, cords, and occasionally gland-like structures. **(B)** Strong, diffuse immunoreactivity for calretinin is observed in the tumor cells. **(C, D)** Tumor cells show positive immunostaining for alpha-inhibin. Original magnification, 100x; scale bars, 200 μm.

In May 2024, the patient underwent a diagnostic hysteroscopy at the Gynecology Department of the Institute for Maternal and Child Health “IRCCS Burlo Garofolo” of Trieste. Uterine cavity was entirely occupied by diffuse polypoid hyperplasia with accentuated and atypical vascularization. A 20 mm antero fundic wall polyp with atypical aspect was present. Polypectomy and multiple biopsies were performed. Histopathology showed flaps of atrophic fibroglandular endometrial polyp with focal morular squamous metaplasia. Due to the discrepancy between the hysteroscopic view and the histological examination an operative hysteroscopy in the operating setting was arranged in September 2024. The exam showed a thickened endometrium with accentuated vascularization. A 15 mm atypical polyp of the posterior wall was found. Polypectomy and endometrial ablation were performed. In one of the pieces analyzed, proliferation of glandular elements with mild nuclear characteristics was found, arranged to form cords, nests or trabeculae (**Figures 2A, B**). On immunohistochemical analysis, the neoplasm showed diffuse positivity for vimentin, alpha inhibin, HMB45, CK18, ER, PR, calretinin, WT1, CD56. Finally, the tumor cells were negative for CD10, desmin, smooth muscle actin, EMA, PAX8, CD34, chromogranin, synaptophysin (**Figures 2C, D**). Ki67 1–2%, MMRp. The morphologic and immunohistochemical results were consistent with the diagnosis of Uterine Tumor Resembling Ovarian Sex Cord Tumor (UTROSCT).

An expert preoperative ultrasound demonstrated a sessile, focal non uniform intracavitary lesion at the fundus measuring 15 × 11 × 12 mm characterized by incomplete and irregular contours, and minimal intralesional and perilesional vascularization (CS2) in the context of a thin endometrium (**Supplementary Video 1**). Magnetic resonance imaging (MRI) and abdominal computed tomography (CT) scan confirmed the ultrasound findings and reported some enlarged internal iliac and obturator lymph nodes. Tumoral markers were negative. Based on these findings, the patient underwent total abdominal hysterectomy and bilateral salpingo-oophorectomy in November 2024. Tumor samples were collected and sent to the histopathology laboratory for analysis. On gross examination of the hysterectomy specimen, the uterine fundus was distorted by multiple subserosal leiomyomas, with additional submucosal and intramural myomatous nodules (largest partially calcified). A slightly raised brown-white endometrial lesion measuring 1.5 cm was identified in the proximal uterine cavity and was subtotally submitted for histology within an overall sampling of 14 cassettes (including cervix, lesion, representative leiomyomas, and grossly unremarkable endometrium). Microscopically, the endometrial lesion was a polypoid neoplasm composed of medium-to-large epithelioid cells arranged predominantly in tightly packed nests and cords with rare gland-like structures, with no myometrial invasion identified. Immunohistochemistry on the hysterectomy specimen confirmed the biopsy immunoprofile described above, supporting a diagnosis of group II UTROSCT and no conventional endometrial stromal tumor component was reported in the examined sections, favoring UTROSCT over ESTSCLE on the available material. Histopathological examination of the lymph nodes showed no evidence of metastatic involvement. Both ovaries were histologically unremarkable.

The patient did not receive any adjuvant therapy. Four months following surgery all tumor markers remained within normal levels. No evidence of recurrence emerged during the gynecological examination and transvaginal ultrasound. The patient was asymptomatic and reported clinical well-being. Written informed consent was obtained from the patient for publication of this case and any accompanying anonymized images.

### 2.1 Exploratory serum proteomics (hypothesis-generating)

We performed the serum proteomic analysis by using high-resolution mass spectrometry (HRMS). Protein identification from trace amounts of serum was achieved through efficient depletion of high-abundance proteins, combined with data-dependent acquisition (DDA) and data-independent acquisition (DIA) strategies (8). The regulation of cellular behavior is modulated by the extracellular matrix (ECM), which provides physical, biochemical, and biomechanical signals (9). Cancer cells release a heterogeneous array of proteins, including ECM components, that are essential for ECM remodeling and for supporting tumor growth. A total of 200 µL of crude serum was incubated with ProteoMiner™ beads for two hours at room temperature. Bound proteins were eluted from the column using a solution containing 4% SDS and 100 mM β-mercaptoethanol. 30 µg of total protein were used for mass spectrometry analysis. Protein separation and detection were carried out using an Ultimate 3000 nanoLC system coupled to an Orbitrap Lumos Tribrid mass spectrometer. DDA raw data were processed using Proteome Discoverer 3.1, while DIA data were analyzed using Spectronaut 19. All samples were analyzed in triplicate to ensure reproducibility.

Our proteomic study compared the patient’s serum protein profile with two other control samples: the first one was obtained from a volunteer without comorbidities (CTRL) and the second belonged to a patient affected by endometrial stromal sarcoma (ESS). Proteomic analysis was conducted using both DDA and DIA acquisition methods.

Given the single-case design (biological n=1 per group) and the use of technical replicates, statistical testing was used only for exploratory prioritization of candidates and should not be interpreted as confirmatory.

**Figure 3:**
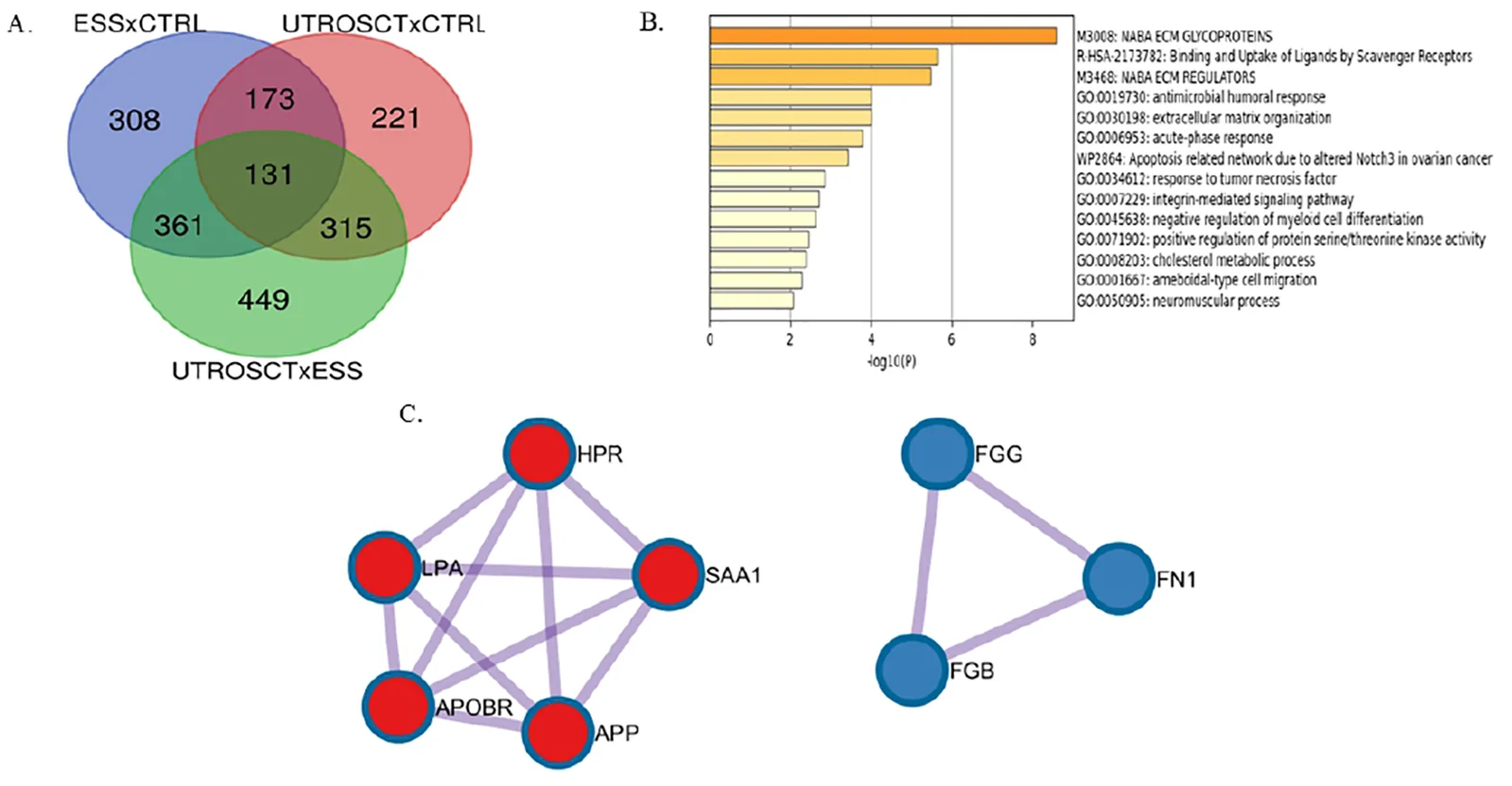
**(A)** Intersection of identified proteins by three different comparisons. **(B)** Overview of the proteomic pathways using the Metascape tool. **(C)** MCODE algorithm applied to identify neighborhoods into which proteins are densely connected.

Three comparisons were performed (ESS vs CTRL, UTROSCT vs CTRL, and UTROSCT vs ESS) for differential expression analysis. Proteins with a fold change ≥1.5 or ≤0.6 and nominal t-test P values <0.05 were considered exploratory candidates for prioritization, given the single-case design and the use of technical replicates. Analyses were conducted using RStudio. Integration of data from both approaches led to the identification of 5,403 unique proteins. The subsequent bioinformatic analysis revealed 131 proteins commonly differentially abundant across all comparisons (**Figure 3**). Pathway enrichment analysis using Metascape tool indicated significant involvement of proteins in extracellular matrix-related pathways, while network analysis using the MCODE algorithm identified a densely connected cluster of proteins: SAA1, HPR, LPA, APP, APOBR, FN1, FGG and FGB.

## 3 Materials and methods

This study was conducted according to the criteria of Preferred Reporting Items for Systematic reviews and Meta-Analyses extension for Scoping Reviews (PRISMA ScR). An *a priori* protocol was developed before study selection and is available on the Open Science Framework (OSF) (https://doi.org/10.17605/OSF.IO/ZE26K).

The review questions were intentionally broad and were formulated using the Population–Concept–Context (PCC) framework. Population: patients with histologically confirmed UTROSCT (including classic UTROSCT and endometrial stromal tumors with sex cord-like elements when explicitly described). Concept: ultrasound descriptors (e.g., ‘fibroid-like,’ ‘polyp-like,’ intracavitary mass, pelvic mass). Context: any clinical setting in which pelvic/transvaginal ultrasound was performed.

### 3.1 Study design, database search and information sources

The research strategy adopted the following key words: “case report” AND “UTROSCT” OR “uterine tumor resembling ovarian sex cord tumors” OR “UTROSCTs”. We performed a comprehensive search from the electronic databases MEDLINE (Pubmed), Scopus and Google Scholar from January 2004 to December 2025 in order to identify case reports regarding UTROSCT. We decided to include this time interval since articles dealing with this tumor prior to this period are extremely rare and the immunohistochemical data are incomplete. We included English-language case reports, including articles with non-English titles but with English full text available, describing histologically confirmed UTROSCT and reporting at least one pelvic/transvaginal ultrasonographic assessment. We reviewed the literature regarding the mean age of patients and the ultrasound patterns of UTROSCT.

### 3.2 Study selection and data analysis

All studies identified were listed by title, authors, and year of publication. Case series were excluded *a priori* to avoid duplicate case ascertainment and because imaging details were frequently aggregated or incompletely reported. We have followed the PRISMA ScR checklist. The study selection process is presented in **Table 1**, using a PRISMA flow diagram adapted for scoping reviews. Two independent investigators screened the title and abstracts based on the predefined eligibility criteria (359 records identified). The same two authors reviewed independently the full text of papers identifying those to be included in the review. Discrepancies were resolved by consensus. 5 manuscripts were excluded for duplication. 101 manuscripts were excluded because they do not include clinical cases. 112 works were excluded because they were off topic. 28 manuscripts were excluded because of language. 14 works were excluded because only the abstract was accessible. In total, 99 cases were ultimately included (11–109).

**Table 1:** Study design.

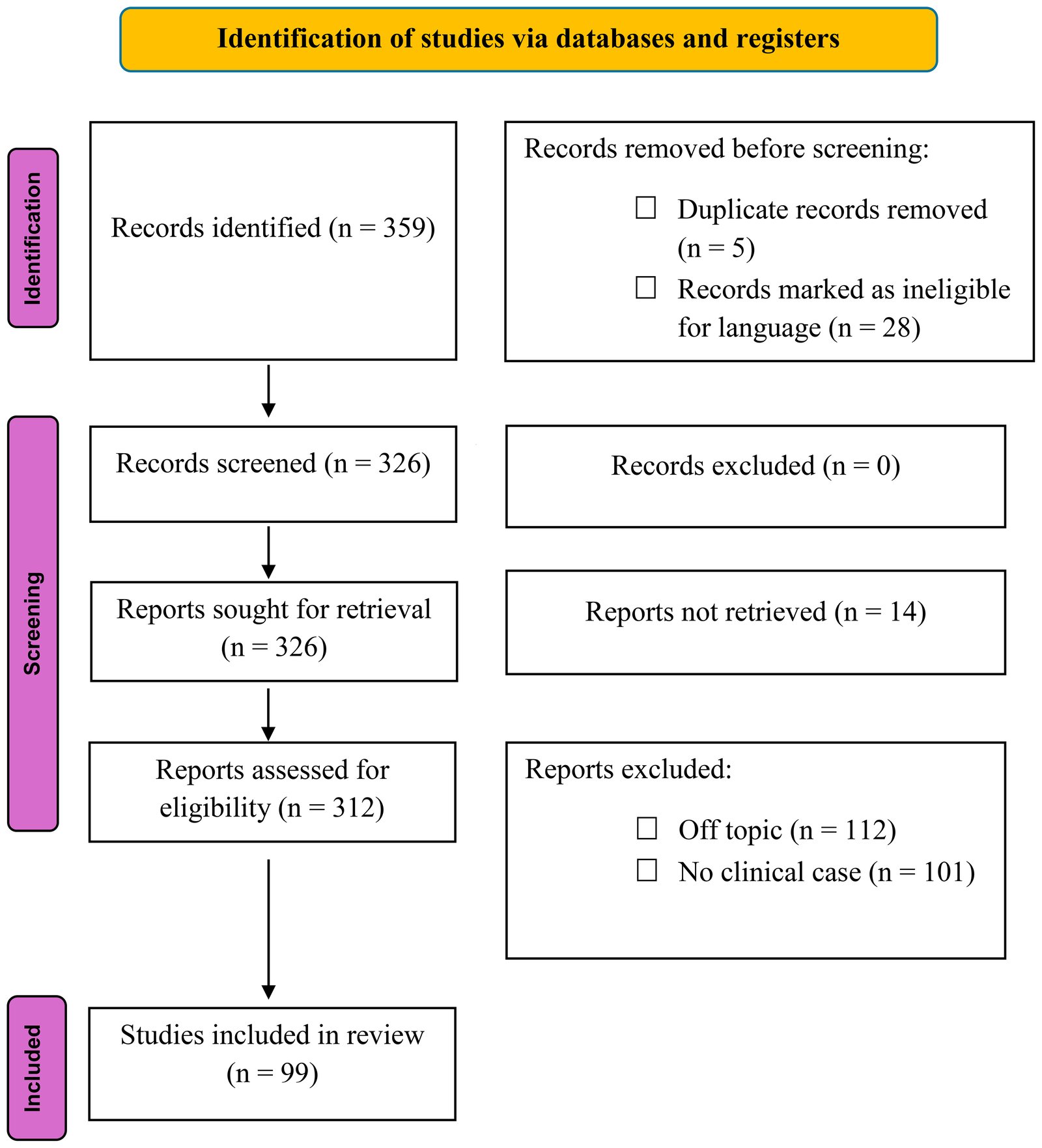

PRISMA 2020 flow diagram (10).

### 3.3 Assessment of methodological quality

The methodological quality of the included studies was assessed using the Joanna Briggs Institute (JBI) Critical Appraisal Checklist for case reports, appraisal results were not used to exclude sources of evidence.

The study was conducted according to the guidelines of the Declaration of Helsinki, and approved by the Institutional Review Board of Institute for Maternal and Child Health IRCCS “Burlo Garofolo”, Trieste (IRB: RC 08/2020–15 April 2020). This case report did not require approval by an institutional ethics committee, as it is based on a retrospective description of a single clinical case managed according to standard diagnostic and therapeutic procedures. All patient data were fully anonymized prior to analysis, and no experimental interventions were performed.

### 3.4 Assessment of risk of bias and limitations

This scoping review is based on case reports, which may be affected by publication and selective reporting bias; therefore, the reported frequencies of ultrasound patterns should be interpreted as a descriptive mapping of published reports rather than as population-level estimates. Because ultrasound descriptions in these reports are often heterogeneous, incomplete, and non-standardized, we did not perform further stratified analyses or infer ultrasound features beyond those explicitly reported, in order to avoid introducing bias. Another limitation is that the majority of the included studies did not specify whether the diagnosed UTROSCT was group I or group II, thereby precluding the distinction of ultrasonographic features between these two categories.

## 4 Results

**Figure 4:**
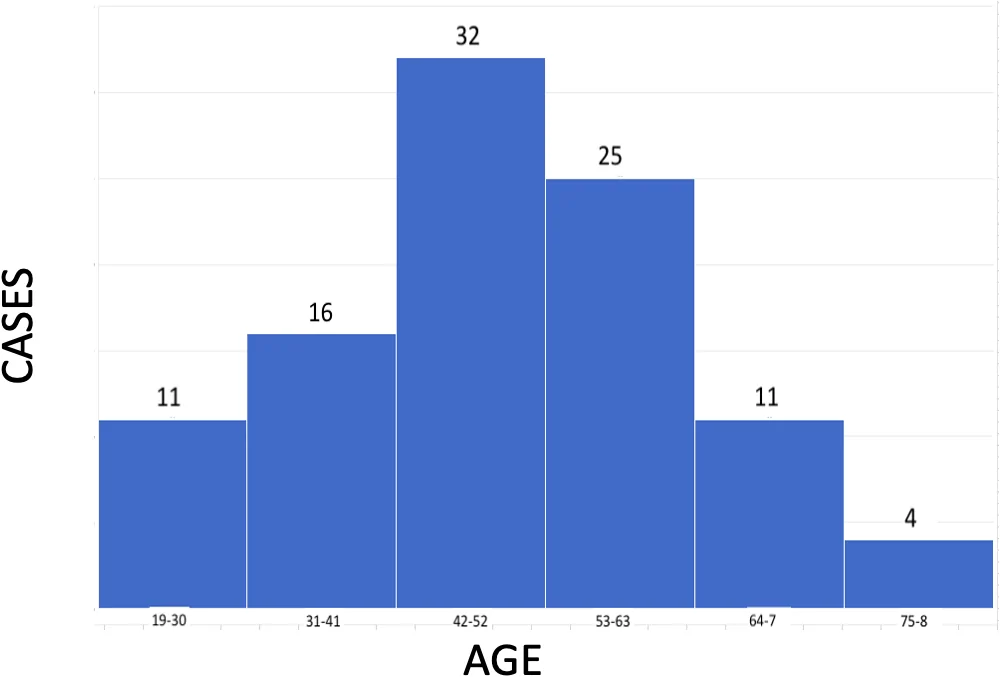
Age distribution of the sample.

The mean age of the examined population is 49.2 years, 24 patients (24.2% of the sample) are younger than 40 years. The younger patient of our sample is 19 years old, the older one is 83 years old. The age distribution is displayed in **Figure 4**.

The results of our review showed that UTROSCT presents as a fibroid in the majority of cases (46.5%), less frequently as a pelvic mass (11.1% of cases) or as an endometrial polyp (10.1% of cases) as reported in **Table 2**. In 32.3% of patients (n=32) ultrasound features are not specified.

**Table 2:** UTROSCT ultrasound patterns.

Indication	n	%
Fibroid	46	46.5%
─ Submucosal	11	11.1%
─ Intramural	8	8%
─ Not specified	27	26.4%
Polyp	10	10.1%
Pelvic mass	11	11.1%
Not specified	32	32.3%
Total	99	

## 5 Discussion

In 1976, Clement and Scully identified an unusual subset of uterine neoplasms exhibiting histological features reminiscent of ovarian sex-cord tumors (4). These cancers were subsequently categorized into two distinct entities: UTROSCTs and ESTSCLE with lower sex cord-like differentiation, characterized by a higher risk of recurrence and metastasis, and considered a separate pathological group (110).

The aim of this manuscript is to analyze the mean age and the ultrasound features of UTROSCT. We also focused on the proteomic pattern of UTROSCT analyzing in our case report the serum protein profile of the patient. Literature data regarding this topic are limited. We purposely present ultrasound as the clinical–radiologic phenotype and serum proteomics as a separate, hypothesis-generating assessment of a potential systemic/ECM-related phenotype. At present, the literature does not support a validated link between specific ultrasound patterns and circulating proteomic signatures in UTROSCT; therefore, we avoid mechanistic inferences and interpret proteomic results cautiously.

In the absence of robust evidence linking sonographic appearance to underlying tumor biology, we propose a tentative, hypothesis-based interpretation of the most recurrent ultrasound descriptors reported in the literature. First, when UTROSCT is described as a “fibroid-like” lesion—particularly as an intramural mass—this likely reflects a predominantly myometrial-centered growth pattern. In molecular series, GREB1-altered tumors have tended to present more often as intramural masses, whereas ESR1-altered tumors have more frequently shown a submucosal/polypoid presentation, suggesting that at least part of the phenotypic variability observed on imaging may track with molecular subtypes (111). Second, a “polyp-like” or intracavitary lesion on ultrasound plausibly corresponds to submucosal/polypoid growth protruding into the uterine cavity, a gross phenotype that falls within the recognized morphologic spectrum of UTROSCT (111). Third, Doppler vascularity is reported inconsistently across case reports and, when described, may reflect differences in stromal composition and vascular density; however, the heterogeneity and incompleteness of reporting preclude unbiased comparisons or any definitive inference regarding clinicopathologic correlates.

Our case involves a postmenopausal woman, even though the mean age emerging from our review is 49.2, demonstrating that premenopausal women represent the main demographic affected by this tumor. In particular the 51.5% of the sample, included in our review, are women younger than 50 years old (n=51). This demographic distribution suggests a potential role of hormonal influence in the tumorigenesis of UTROSCTs, warranting further investigation.

In support of this hypothesis, characteristic gene fusions involving *ESR1* and *GREB1*, key components of estrogen signaling pathways, have been identified in these tumors (11). Moreover, hormone receptors, particularly estrogen receptor (ER) and progesterone receptor (PR), are expressed in the majority of UTROSCTs (70–100%) (1, 112). These findings highlight the importance of routinely assessing hormone receptor status as part of the diagnostic workup. Given the potential hormone sensitivity of these neoplasms, hormonal therapy may represent a rational, targeted therapeutic approach in receptor-positive cases. In light of the rarity of UTROSCTs and the current absence of standardized treatment protocols, evaluating the hormonal profile could provide essential insights for developing more individualized and effective management strategies (113, 114).

The most frequent clinical presentation consisted of abnormal uterine bleeding, while pelvic pain or aspecific abdominal discomfort were reported less frequently. In a subset of patients, the tumor remained asymptomatic and was identified incidentally during routine gynecologic examinations or infertility assessments (12–14).

Ultrasonography can be used as a screening test because it is less expensive, easily available and not invasive (15). As reported by E.A. Blake et al. in their systematic review published in 2014, ultrasonography is commonly used for preoperative imaging (53.5%), instead of that, computed tomography (CT) and magnetic resonance imaging (MRI) are adopted less frequently (14.0%) (5).

To date, no studies have been published regarding only the ultrasound manifestation of UTROSCT. The most common ultrasound features are usually suggestive of uterine leiomyoma. UTROSCTs are frequently misidentified as leiomyomas due to their typical submucosal or intramural growth patterns. As described by Watrokski et al. in their review published in 2024 in about 15% of cases (8% in our review), these neoplasms look like polypoid lesions, protruding into the uterine cavity. These tumors lack specific radiological features on ultrasound or MRI. Although some MRI-based descriptions are available in the literature, none have led to a definitive diagnosis prior to histopathological evaluation (16, 115).

The regulation of cellular behavior is modulated by the ECM, which provides physical, biochemical, and biomechanical signals (9). Cancer cells release a heterogeneous array of proteins, including ECM components, that are essential for ECM remodeling and for supporting tumor growth. Shotgun proteomics is able to identify a large number of low abundance serum proteins by combining the two most commonly used approaches, DDA and DIA (116). In this study, proteins related to ECM were identified using advanced proteomics techniques and serum depletion. The ECM is a highly dynamic structure that plays an essential role in tissue homeostasis (117). ECM molecules are responsible for regulating all the characteristics of cancer, including initiation and metastatic progression, and are released into the bloodstream during tumorigenesis (118). The glycoproteins component of ECM play a key role in cell migration and proliferation (119). FGG and FGB are components of ECM and play an important role in the modulation and controlling of cancer progression (120). FN1 is another component of ECM that acts as a key protein in the communication between the intra and the extracellular environment (121). In summary, ECM proteins modulate the cancer progression from the beginning to metastatic development and are released continuously into the bloodstream during tumorigenesis.

Interestingly, the results of our proteomic analysis diverge from what is commonly reported in the medical literature regarding the clinical behavior of this tumor type. UTROSCTs are generally classified as having low malignant potential, due to their low rates of metastasis and recurrence. Our data, as previously reported, suggest the possibility of a more heterogeneous biological profile. This discrepancy highlights the need for further studies to reassess the prognostic assumptions currently associated with these neoplasms. However, these proteomic observations should be interpreted with caution. The exploratory serum proteomics component is based on an N-of-1 biological design and lacks tumor-tissue immunohistochemical validation of the mass spectrometry–identified targets, which limits biological attribution (tumor vs microenvironment vs systemic response) and clinical interpretability. Given the N-of-1 design and potential analytical variability, proteomic findings should be considered hypothesis-generating and require validation in independent cohorts.

The majority of patients undergo hysterectomy and/or salpingo-oophorectomy. Lymph node dissection is generally not performed. Lymphoadenectomy is recommended only in the presence of ovarian or lymph node metastases identified on preoperative imaging. As emerging from our research, confirmed by the systematic review of Mami Shibahara et al, which report that patients younger than 40 years old are less than 25% of the UTROSCT population, it is mandatory to consider fertility-preserving treatment (17).

Nevertheless, long-term post-treatment follow-up is essential, as recurrence or distant metastasis have been reported several years after initial therapy. Group I tumors (ESTSCLE) are generally characterized by benign course, despite they are considered to have low-grade malignant potential; in fact rare cases of recurrence are described in literature, which occur almost exclusively in this subtype. The overall recurrence rate remains low, approximately 5%, with a median time to recurrence of 24 months. Regular follow up is recommended, at least every six months (5, 115).

## 6 Conclusion

UTROSCT is extremely rare; available registry-based and derived estimates suggest an annual incidence in the order of magnitude of one case per several million women. For diagnostic purposes, ultrasound is the preferred modality, and the typical presentation resembles that of a uterine fibroid. The standard treatment involves hysterectomy with or without adnexectomy; however, fertility-sparing options are available for younger patients. The proteomic analysis identified the presence of FGG, FGB, and FN1, which modulate and regulate cancer progression processes, although this seems to contrast with the biological behavior of UTROSCT. The exploratory serum proteomics findings should be interpreted cautiously, these results are hypothesis-generating only and require replication and orthogonal validation in independent cohorts. Recurrence rates are low (approximately 5%), with a higher prevalence in group I tumors. Given its rarity and the limited case series, further studies are warranted.

## Supplementary material

Supplementary Video 1 Sonographic appearance of UTROSCT after hysteroscopy.
