# Correction: UTROSCT: a case report and scoping review of ultrasound features, with exploratory serum proteomic findings

**DOI:** 10.3389/fonc.2026.2035290

**Published:** 2026-10-28

**Authors:** Davide Castelli, Ghergana Alexandrova Topouzova, Blendi Ura, Francesca Buonomo, Eduardo Maria Sommella, Alessandro Mangogna, Federico Romano, Giovanni Di Lorenzo, Stefania Cicogna, Andrea Romano, Giuseppe Ricci

**Affiliations:** 1 Department of Medicine, Surgery and Health Sciences, University of Trieste, Trieste, Italy; 2 Institute for Maternal and Child Health, IRCCS Burlo Garofolo, Trieste, Italy; 3 DIFARMA Department, University of Salerno, Salerno, Italy; 4 Pathology (UCO Anatomia Patologica), Cattinara Teaching Hospital, Trieste, Italy

**Keywords:** case reports, proteomics, scoping reviews, sex cord-gonadal stromal tumors, ultrasonography, uterine neoplasms

In the published article, a correction has been made to Section 1, **Introduction** (paragraph containing the annual incidence estimate):

The original sentence reads:

“Based on which allows for an estimated annual incidence of UTROSCT in Europe of 0.00003% (1 womanper Eurostat data from 2024, as of January 2023, there were 229 million women in Europe, 3 million/year).”

And is replaced with the following revised version:

“Based on Eurostat data published in 2024, the female population in Europe was 229 million as of January 2023, corresponding to an estimated annual incidence of UTROSCT of 0.00003% (approximately 1 case per 3 million women per year in Europe).”

The original version of this article has been updated.

